# Identification of a G2-like transcription factor, OsPHL3, functions as a negative regulator of flowering in rice by co-expression and reverse genetic analysis

**DOI:** 10.1186/s12870-018-1382-6

**Published:** 2018-08-06

**Authors:** Liping Zeng, Xue Liu, Zhuangzhi Zhou, Dayong Li, Xianfeng Zhao, Lihuang Zhu, Yingfeng Luo, Songnian Hu

**Affiliations:** 1 CAS Key Laboratory of Genome Sciences and Information, Beijing Institute of Genomics, Chinese Academy of Sciences, NO.1 Beichen West Road, Chaoyang District, Beijing, 100101 China; 2 State Key Laboratory of Plant Genomics and National Center For Plant Gene Research, Institute of Genetics and Developmental Biology, Chinese Academy of Sciences, NO.1 Beichen West Road, Chaoyang District, Beijing, 100101 China; 3 University of Chinese Academy of Sciences, No.19(A) Yuquan Road, Shijingshan District, Beijing, 100049 China

**Keywords:** Co-expression, Flowering time, *OsPHL3*, MYB-CC transcription factor, G2-like, Rice

## Abstract

**Background:**

Flowering time is a key trait for regional adaption and seed production in rice (*Oryza sativa* L.). Forward and reverse genetic studies have characterized a number of flowering-time genes. However, co-expression analysis has not been used to identify the flowering-time genes.

**Results:**

We predicted a G2-like family transcription factor, *OsPHL3*, by co-expression networks analysis with photoperiodic flowering pathway genes. OsPHL3 contains a MYB-CC domain, and was localized in the nucleus with transcriptional activation potential. *OsPHL3* was mainly expressed in the leaves and exhibited a circadian rhythmic expression pattern. Rice lines overexpressing *OsPHL3* showed a delayed flowering time in the genetic background of TP309 under both long-day (Beijing) and short-day (Hainan) conditions. By contrast, the knockout rice lines of *OsPHL3* by CRISPR/Cas9 technology promoted flowering time regardless of genetic backgrounds (i.e. Nipponbare and TP309) or day length. Further analysis indicated that *OsPHL3* delayed flowering time by down-regulating the expression of *Hd3a* and *RFT1* through promoting *Hd1* under long-day conditions (LDs), or suppressing *Ehd1*/*Hd1* under short-day conditions (SDs).

**Conclusions:**

Our results suggested that co-expression analysis is a useful strategy for identifying novel flowering-time genes in rice.

**Electronic supplementary material:**

The online version of this article (10.1186/s12870-018-1382-6) contains supplementary material, which is available to authorized users.

## Background

Flowering time is one of the most important agronomic traits in determining grain yield and regional adaptation [1–3]. In rice, there are two independent pathways controlling photoperiodic flowering. The first one is *OsGI-Hd1-Hd3a* pathway [4–6], which is conserved between *Arabidopsis thaliana* and rice [7]. The second one is the *Ghd7-Ehd1-Hd3a/RFT1* pathway, which is unique in rice [6, 8–10]. The florigen proteins, *Hd3a* and *RFT1*, work as integrated mobile flowering signals, and move from leaf to the shoot apical meristem to induce reproductive development [11, 12].

The rice *OsGI-Hd1-Hd3a* pathway has a one to one correspondence with *Arabidopsis GIGANTEA* (*GI*) – *CONSTANS* (*CO*) - *FLOWERING LOCUS T* (*FT*) [7]. *OsGI* promotes the expression of *Hd1*, but *Hd1* has different roles under LDs and SDs [5]. Under short day conditions (SDs), *Hd1* activates *Hd3a* expression, while under long day conditions (LDs), it represses *Hd3a* expression [6, 7]. The functional conversion of *Hd1* is determined by interacting with the *phytochrome B* (*phyB*)-mediated signaling [13]. For the *Ghd7-Ehd1-Hd3a/RFT1* pathway that is unique to rice, *Grain number, plant height, and heading date 7* (*Ghd7*) acts as upstream of *Early heading date1* (*Ehd1*), which does not have an ortholog in *Arabidopsis*. Under SDs, *Ehd1* expression is induced by the products of *Ehd2* [14], *Ehd3* [15], *Ehd4* [16] and *OsMADS51* [17], but repressed by *OsCOL4* [18]. Under LDs, *Ehd1* expression is upregulated by *Ehd2* [14], *Ehd4* [16] and *OsMADS51* [17], downregulated by *OsCOL4* [18], *OsCOL10* [19] and *DTH8* [20].

Until now, most of the flowering-time genes were identified by map-based cloning in mutant plants or plants with natural variations, or alternatively by overexpression and knockdown transgenic plants [1]. For example, the function of *OsMADS34* [21] and *OsTrx1* [22] was investigated by knockdown transgenic lines; while that of *OsMADS15* [23], *OsMADS50* [24, 25], *OsMADS56* [25], *OsMYB1R1* [26], *OsBBX14* [27] and *OsCOL13* [28] was revealed by overexpression transgenic lines. Co-expression analysis in rice has been proven to be a useful tool for identifying the function of transcription factors, such as *HYR* in regulating the photosynthesis process and *RSR1* in controlling starch biosynthesis [29, 30]. However, the co-expression analysis has not been used to predict the flowering-time genes in rice.

Here, we conducted co-expression networks analysis in rice to interrogate genes that can play roles in flowering-time. Our study identified a G2-like family transcription factor containing Myb-CC domain that we referred to as OsPHL3. Functional characterization revealed that OsPHL3 plays an important role in regulating rice flowering time.

## Results

### Gene co-expression analysis identified G2-like family transcription factors involving in flowering time pathway in rice

In plants, leaves perceive information of day-length and temperature changes, which was subsequently used to determine flowering time [31, 32]. The mobile flower-promoting signal molecules, Hd3a and RFT1, are generated in leaves and transferred to the shoot apical meristem to induce flowering [11, 12]. We used a published leaf transcriptome data [33] to predict flowering time regulators in rice.

The key rice flowering time genes, including *OsGI*, *Ghd7*, *Hd1*, *Ehd1*, *RFT1*, *Hd3a*, *OsMADS14* and *OsMADS15*, were selected as “guide genes” to identify the co-expressed genes using leaf transcriptome data.

Among the candidate genes involved in flowering time regulation, we were particularly interested in transcription factors, because they accounted for 82% of known flowering-time genes [1] .We calculated absolute value of the Pearson correlation coefficient and selected those greater than 0.8 between the flowering time regulators and TFs (Additional file 1: Table S1). We illustrated the top five most abundant TF families with member genes of co-expression to flowering time regulators (Additional file 1: Table S1). Among these TFs, the shared top-ranked co-expressed TFs were the member of G2-like family (Additional file 1: Table S1; Additional file 2: Figure S1).

**Fig. 1:**
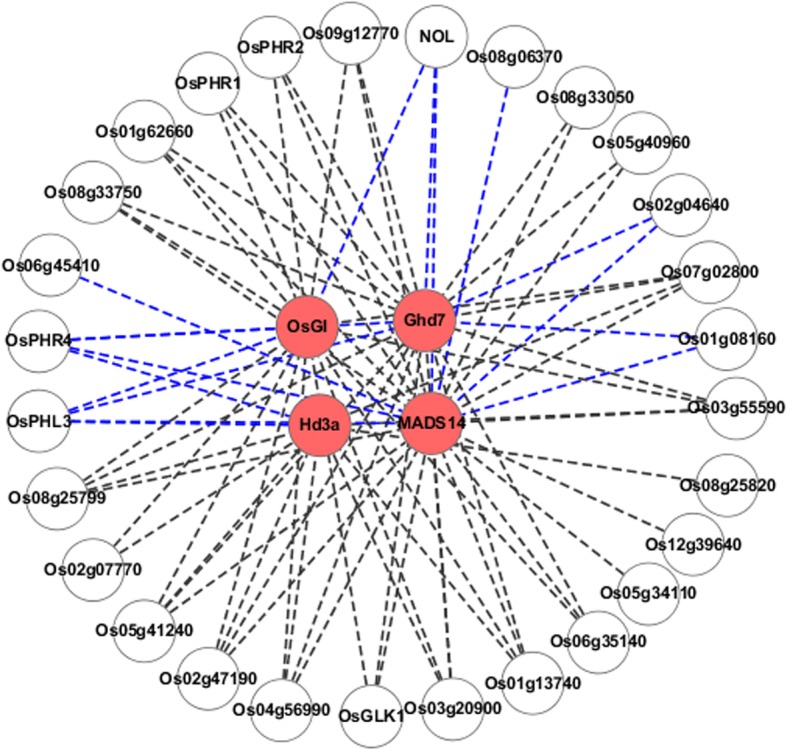
Co-expression networks of rice G2-like transcription factors and flowering time regulators. Co-expression network between flowering time regulators and G2-like transcription factors based on a Pearson correlation coefficient cutoff of 0.6. Nodes in the maps represent genes. Red node represents the flowering- time genes in photoperiodic pathway. The blue lines connecting two genes represent transcription factors negatively co-expressed with photoperiodic flowering pathway genes. Black lines connecting two genes represent transcription factors positively co-expressed with photoperiodic flowering pathway genes

With the absolute value of Pearson correlation coefficient greater than 0.6, 28 G2-like family transcription factors were co-expressed with flowering time pathway genes (Fig. 1). Of those genes, 21 genes were positively co-expressed and 7 genes were negatively co-expressed. Negative regulation of flowering-time genes increase grain yield of rice [9, 26, 35, 36]. Therefore, to identify negative regulators of flowering time are of agricultural importance. We were intrigued by one of the identified TFs, LOC_Os09g12750, which displays negatively correlated co-expression with many flowering regulators including *OsGI*, *Ghd7*, *Hd3a* and *OsMADS14.* These results led us to the hypothesis that LOC_Os09g12750 was a new negative regulator of flowering time. We chose LOC_Os09g12750 for further studies, and referred to it as OsPHL3 (see below).

### Features of *OsPHL3*

**Fig. 2:**
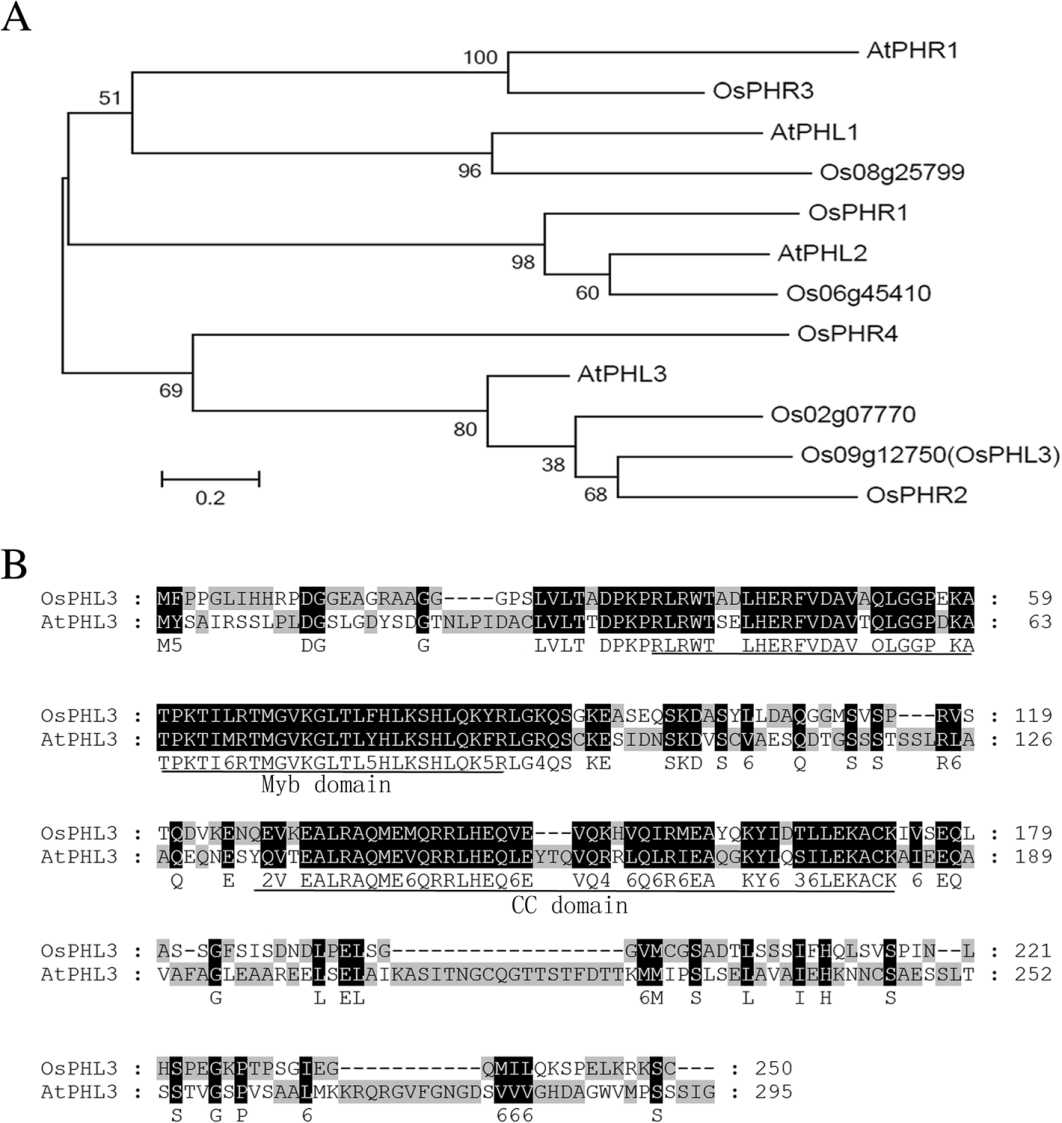
OsPHL3 is closely homologous to AtPHL3 in *Arabidopsis thaliana* and share a conserved MYB-CC domain. **a** Phylogeny of OsPHL3 and related MYB-CC proteins between rice and *Arabidopsis*. An un-rooted tree was constructed by the sequences of MYB DNA-binding domain and CC domain. **b** Protein sequence alignment between OsPHL3 and AtPHL3. The conserved Myb DNA-binding domain (35–86) and CC domain (127–174) were underlined

According to the Rice Genome Annotation Project Database (http://rice.plantbiology.msu.edu), LOC_Os09g12750 encodes a G2-like family transcription factor with a length of 250 amino acids and a molecular mass of approximately 27 KD. The pfam database shows that the protein has one Myb DNA-binding domain (35–86) and one CC domain (127–174) (http://pfam.xfam.org/search/sequence). As revealed by phylogenetic analysis of homologous proteins between rice and *Arabidopsis*, LOC_Os09g12750 is closely related to AT3G24120 (*AtPHL3*, *PHR1 like3*) (Fig. 2a). Protein sequence alignment of OsPHL3 and AtPHL3 showed that they were highly conserved in the Myb and CC domains (Fig. 2b). We hereby named LOC_Os09g12750 as *OsPHL3*.

### Expression pattern of *OsPHL3*

We used quantitative real-time PCR (qRT-PCR) to determine the expression level of *OsPHL3* mRNA in various rice tissues and organs. The results showed that *OsPHL3* was ubiquitously expressed in root, culm, leaf, young panicle and mature panicle. The expression level of *OsPHL3* was highest in leaves in both rice varieties of Nipponbare and TP309 (Additional file 3: Figure S2).

Interestingly, we detected a diurnal expression pattern of *OsPHL3* by quantifying the relative abundance of its expression in young leaves in the rice varieties of TP309. The *OsPHL3* transcript was much more abundant at dawn and midnight under both controlled long day (CLD) and controlled short day (CSD) conditions (Additional file 3: Figure S2), suggesting that *OsPHL3* was expressed in a diurnal manner in rice.

### OsPHL3 is localized in the nucleus and has transcriptional activation potential

**Fig. 3:**
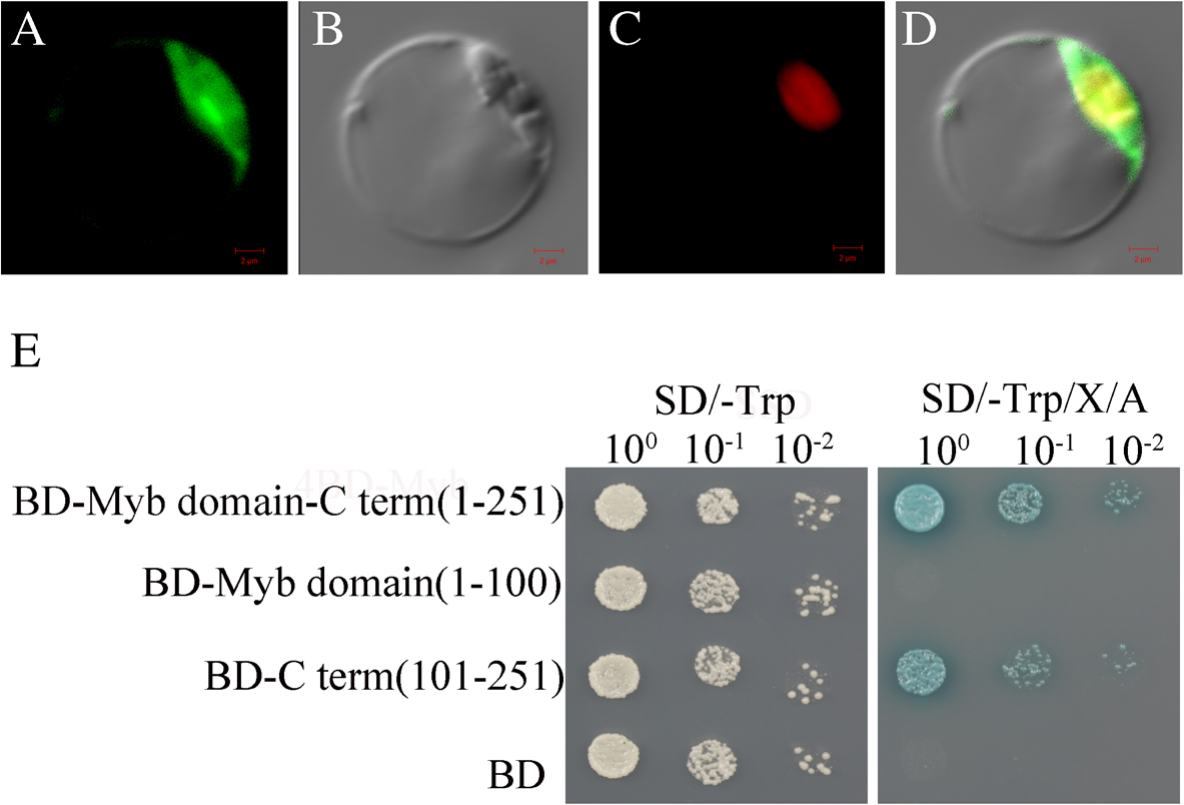
Subcellular localization and transactivation analysis of OsPHL3. **a** Subcellular localization of OsPHL3-GFP fusion protein under the control of CaMV35S promoter. **b** Bright field. **c** The nuclear marker mCherry-VirD2NLS vector (mCherry). **d** Merged image. Bar = 2um from A to D. (E) *OsPHL3* full-length coding region, N-terminal region containing Myb-DNA binding domain (1–100 amino acids) or the C-terminal region containing CC binding domain (101–251 amino acids) were individually inserted into pBD-GAL4 DNA binding-domain contained plasmid. The empty pBD vector was used as control. The expression of *OsPHL3* coding region and *OsPHL3*-C terminal showed resistance to toxic drug Aureobasidin A

We assayed the subcellular localization of OsPHL3. We fused the OsPHL3 with green fluorescent protein (GFP) driving by the cauliflower mosaic virus 35S promoter. The OsPHL3 protein localized predominantly in the nucleus (Fig. 3a-d), suggesting that OsPHL3 likely conducts nuclear function.

To examine if OsPHL3 acts as transcriptional activator, we fused full and partial coding region of OsPHL3 with GAL4 DNA binding domain and transformed into yeast. The results showed that the BD-OsPHL3 (1–251) and BD-C terminal (101–251) fusion protein had transcriptional activation. We speculated that OsPHL3 had the potential to activate transcription in yeast, and that CC domain was required for transcriptional activation (Fig. 3e).

### The *OsPHL3*-OE delayed rice flowering time under LD and SD conditions

**Fig. 4:**
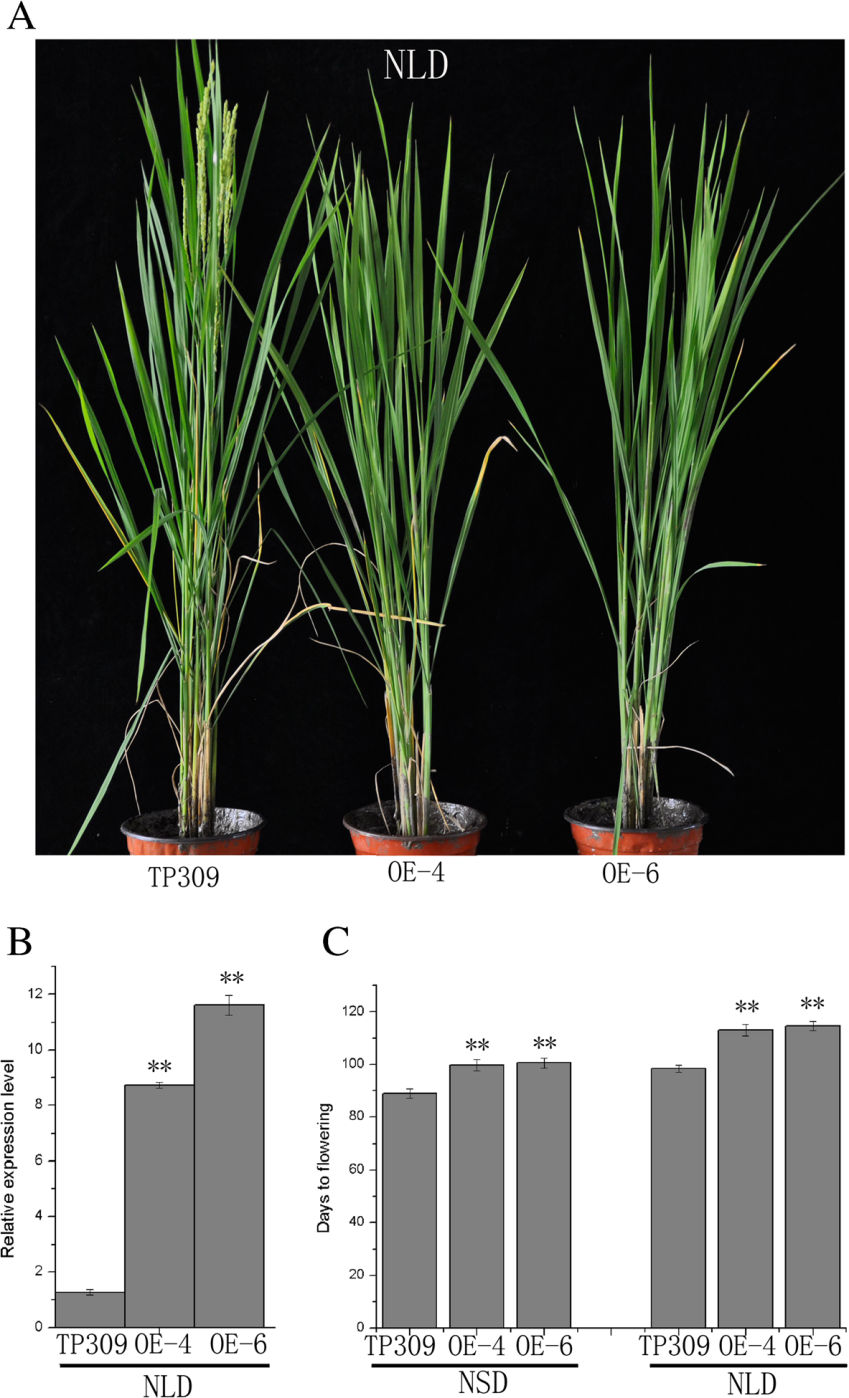
*OsPHL3* overexpression lines delayed flowering. **a** Phenotype characteristics of WT and *OsPHL3*-OE, 107-day-old plants, generated from TP309 genetic background under natural long day (NLD; Beijing). **b** The expression level of WT control and *OsPHL3*-OE (the overexpression lines 4 and 6). **c** Flowering time of WT control and *OsPHL3*-OE under NLDs (Beijing) and NSDs (Hainan) by Student’s t test (**p < 0.01)

To determine whether *OsPHL3* is involved in the regulation of flowering time, we placed full-length coding region of *OsPHL3* into the constitutive cauliflower mosaic virus 35S (CaMV35S) promoter driven over-expression vector and transformed the constructs into the rice variety TP309 by *Agrobacterium*. We obtained 12 independent T0 transgenic *OsPHL3* overexpression (*OsPHL3*-OE) plants. In T1 families, the transgene-positive plants flowered later than the transgene-negative plants. Two overexpression homozygous lines (OE-4 and OE-6) from T2 progeny had a higher expression level of *OsPHL3* in leaves and delayed flowering time (Fig. 4a-b). On average, *OsPHL3-*OE transgenic plants showed a significantly delayed flowering time than WT controls, either by 16 days under natural long day conditions (NLDs, Beijing), or 12 days under natural short day conditions (NSDs, Hainan) (Fig. 4c). These findings support our hypothesis that *OsPHL3* is a negative regulator of flowering time.

### Knockout of *OsPHL3* caused early flowering in long-day and short-day conditions

**Fig. 5:**
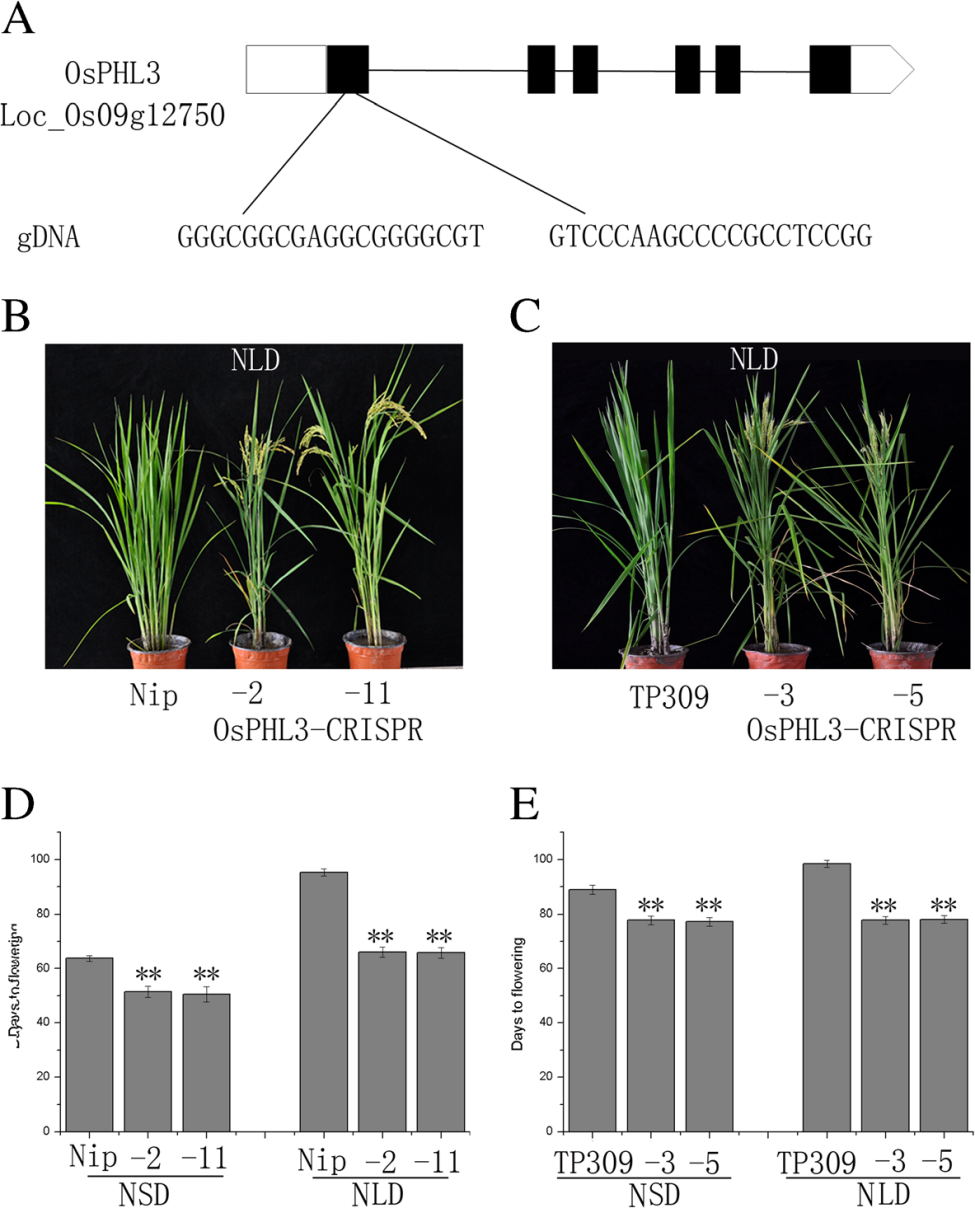
The phenotypic characterization of *OsPHL3* knockout lines. **a** Two target sites for knocking out *OsPHL3* by the CRISPR/Cas9 system. **b** Phenotype of *osphl3* mutants, 78-day-old plants, generated from Nipponbare genetic background under natural long day (NLD; Beijing). **c** Phenotype of *osphl3* mutants, 86-day-old plants, generated from TP309 genetic background under natural long day (NLD; Beijing). **d** Statistical data for flowering time of Nipponbare and *osphl3* mutants under NLDs (Beijing) and NSDs (Hainan). **e** Statistical data for flowering time (**p < 0.01; Student’s t test) of TP309 and *osphl3* mutants under NLDs (Beijing) and NSDs (Hainan)

In order to further validate the function of *OsPHL3* in flowering time regulation, *osphl3* mutants were constructed using the CRISPR/Cas9 technology. We selected two unique sequences in CDS region (36–54 nt and 92–111 nt) as targets for Cas9 cleavage (Fig. 5a), and generated multiple mutant lines. Two homozygous knockout lines of *OsPHL3* (*OsPHL3*-CRISPR-3 and *OsPHL3*-CRISPR-5) were obtained from TP309 genetic background. *OsPHL3*-CRISPR-3 contains a Cytosine insertion at the position of 52 bp of CDS and *OsPHL3*-CRISPR-5 has a 2-bp deletion beginning at the position of 107 bp. We also created *osphl3* mutants using the same method in Nipponbare, a day-length-sensitive cultivar, and successfully obtained two homozygous knockout lines (*OsPHL3*-CRISPR-2 and *OsPHL3*-CRISPR-11). Of these, *OsPHL3*-CRISPR-2 contains an inserted Thymidine at the position of 107 bp of CDS and *OsPHL3*-CRISPR-11 contains an inserted Cytosine at the position of 109 bp.

Under NLDs (Beijing), *osphl3* mutants of TP309 promoted flowering by 21 days than WT on average, while under NSDs (Hainan) *osphl3* knockout lines promoted flowering by 18 days than WT on average. The knockout lines of *OsPHL3* of Nipponbare promoted flowering by 26 days than WT on average under NLDs (Beijing), while promoted flowering by 13 days than WT on average under NSDs (Hainan) (Fig. 5b-e).

Taken together, under both NLD (Beijing) and NSD (Hainan) conditions, knockout lines of *OsPHL3* displayed an early-flowering phenotype in TP309 and Nipponbare. These data strongly support that *OsPHL3* acts as a negative regulator of flowering time.

### Expression of photoperiodic flowering pathway genes in the *OsPHL3*-OE plants under LD and SD conditions

To investigate the functional mechanism of *OsPHL3* in photoperiod flowering pathway, we compared the expression pattern of six flower time genes (*OsGI*, *Ghd7*, *Hd1*, *Ehd1*, *RFT1* and *Hd3a*) in *OsPHL3*-OE and WT control under CLD and CSD conditions.

**Fig. 6:**
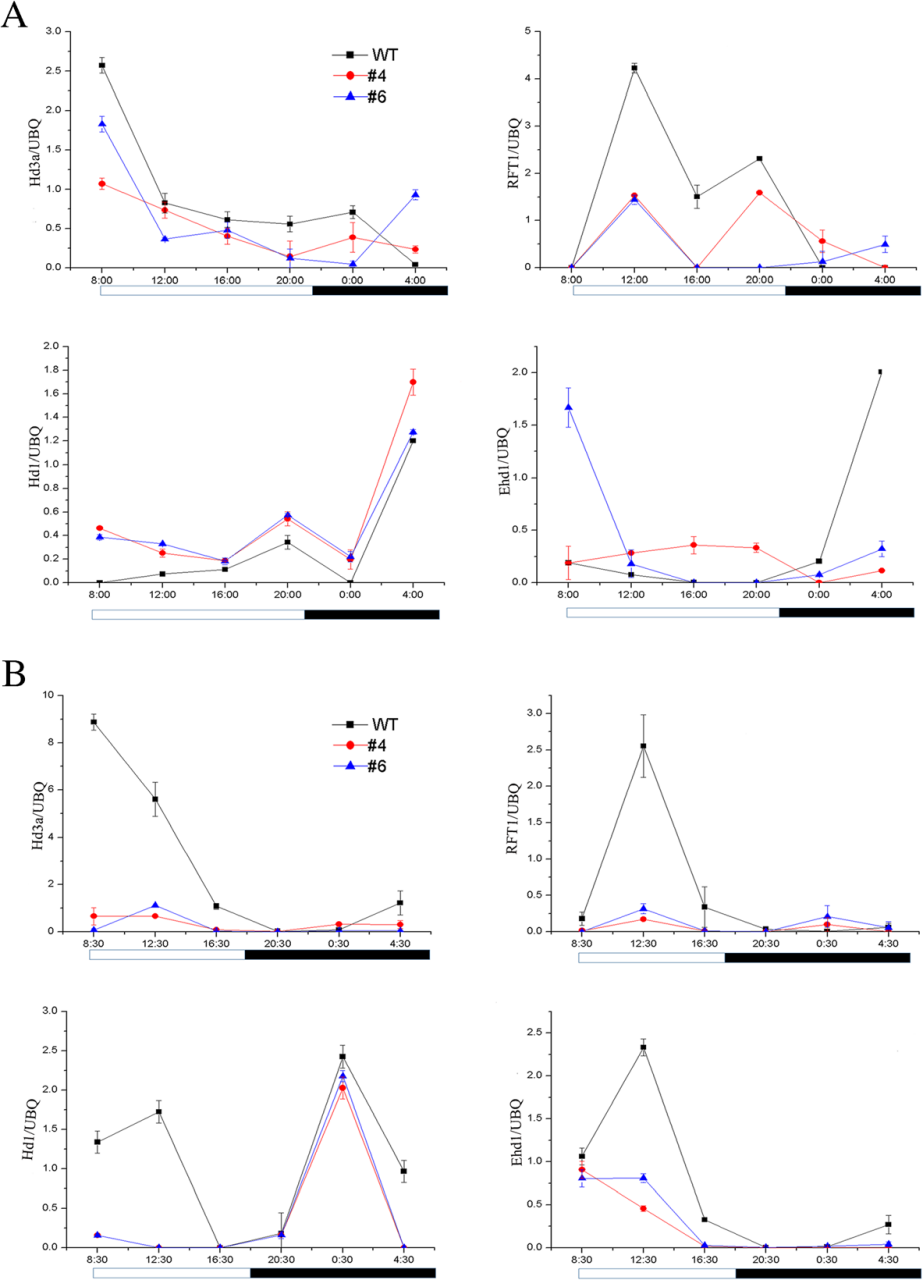
Diurnal expression pattern of key floral regulators between WT and OsPHL3-OE transgenic plants. **a-b** Real time PCR revealed the transcript levels of *Hd3a*, *RFT1*, *Hd1* and *Ehd1* under LDs (**a**) and SDs (**b**). *Ubiquitin* gene of rice was used as internal control. The black lines represent the WT. Red and blue lines represent *OsPHL3*-OE4 and *OsPHL3*-OE6 transgenic plants, respectively. White bars indicate the light period, and black bars indicate the dark periods. The numbers below the bars represent the hours of the day. The mean of each point is based on the average of three technical repeats and two biological repeats

In rice, the florigen genes, *Hd3a* and *RFT1*, are crucial regulators of the transition from vegetative to reproductive phase. Thus, we firstly investigated the expression level of *Hd3a* and *RFT1* in WT (TP309) and *OsPHL3-*OE lines. For all time points, the expression levels of *Hd3a* and *RFT1* in WT control were much higher than in *OsPHL3-*OE lines under CLD and CSD conditions (Fig. 6). This result indicated that *OsPHL3* functioned in the upstream of *Hd3a* and *RFT1*.

Two other genes, *Hd1* and *Ehd1*, are responsible to integrate a network of genes to control the expression levels of florigen genes, *Hd3a* and *RFT1*, and to control flowering time. Therefore, to detect whether *OsPHL3* regulates *Hd3a* and *RFT1* though *Hd1* and *Ehd1,* we examined the expression levels of *Hd1* and *Ehd1* in *OsPHL3-*OE lines and WT (TP309). Under controlled long day conditions (CLDs), *Hd1*, which functions as a negative regulator of florigen under LDs, was expressed at a higher level in *OsPHL3-*OE lines than in WT control in all sampled time points, while Ehd1 didn’t exhibit consistent expression manner (Fig. 6a). Under controlled short day conditions (CSDs), the expression of *Ehd1* and *Hd1* were constitutively repressed in the *OsPHL3-*OE lines compared with WT (Fig. 6b). As controls, the expression level of *GI* and *Ghd7* were also tested, but we found no difference of them between *OsPHL3*-OE and control (TP309) (Additional file 4: Figure S3)*.*

**Fig. 7:**
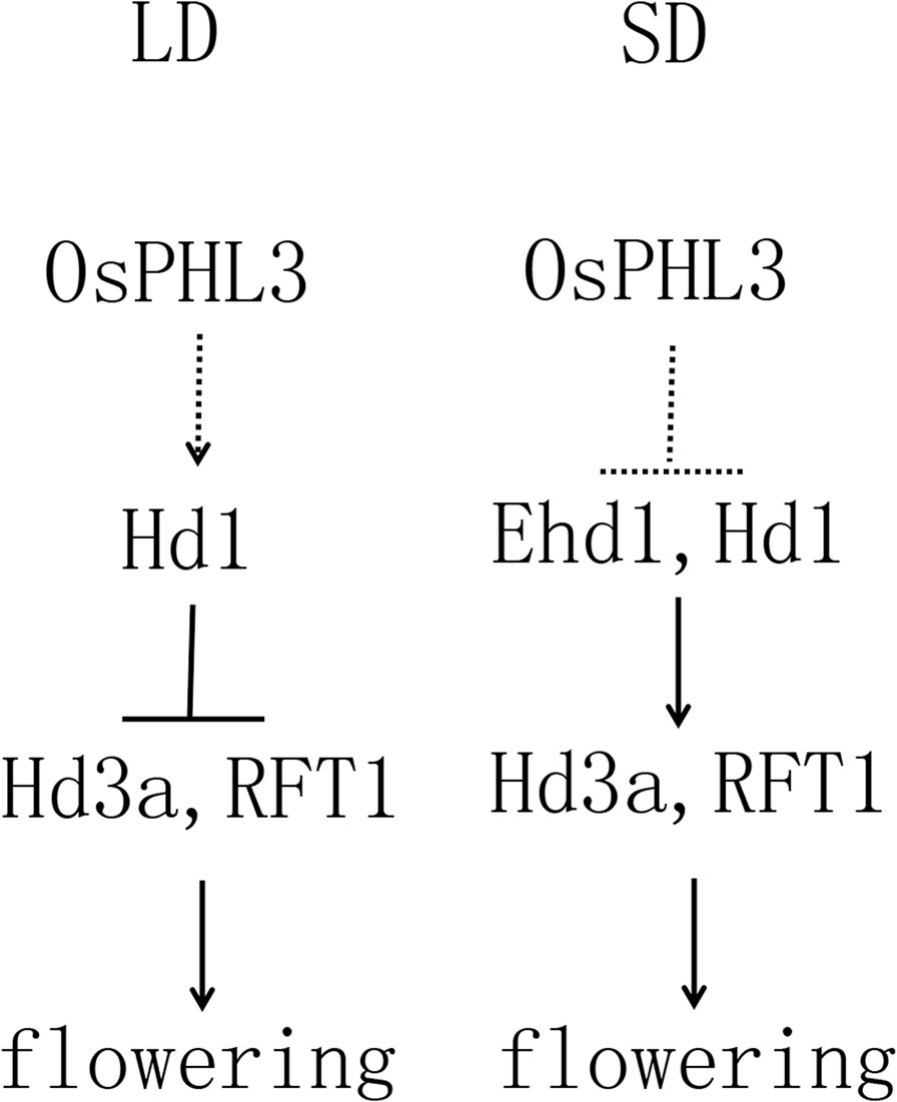
The proposed model for *OsPHL3*-mediated flowering pathway under LD and SD conditions in rice. Under LDs, *OsPHL3* delayed flowering time by downregulating expression of *Hd3a, RFT1* through promotion of *Hd1*. Under SDs, *OsPHL3* may repress the expression of *Hd3a*, *RFT1* by repression of *Ehd1/Hd1*

Taken together, the late-flowering phenotype of *OsPHL3*-OE plants may be at least partially caused by the down-regulation of *Hd3a* and *RFT1* expression. Under SDs this was achieved through repression of *Ehd1/Hd1*, whereas under LDs it was generated by promotion of *Hd1* (Fig. 7).

## Discussion

### Gene co-expression networks can be useful for identifying flowering time genes

Gene co-expression analysis is based on the assumption that genes with similar expression pattern may be functionally related [34]. This approach has been used for predicting the function of uncharacterized genes involved in many biological processes in plants, such as *Arabidopsis* glucosinolate biosynthesis [35], wheat spike architecture regulation [36], the tomato fruit ripening [37] and soybean oil synthesis [38]. In rice, gene co-expression networks successfully predicted the function of *RSR1* (Rice Starch Regulator1) in starch biosynthesis [30] and *HYR* (HIGHER YIELD RICE) in photosynthesis process [29]. In this study, we identified a flowering time regulator *OsPHL3* by gene co-expression analysis. Our results revealed that gene co-expression analysis was a powerful tool for predicting gene function from transcriptome data.

### G2-like family transcription factors are also involved in the regulation of rice flowering

Golden2-like (G2-like) are members of GARP superfamily transcription factors [39]. OsPHL3 belongs to G2-like family transcription factor containing MYB-CC domain. In rice, the G2-like transcription factors participate in chloroplast formation, nutrient assimilation, as well as stress and disease resistance. One of the G2-like members *OsGLK* is a key regulator of chloroplast development and also plays a role in resistance to pathogen invasion [37]. Another G2-like factor *SLL1* regulates leaf rolling [38]. Additionally, the *NIGT1* is a nitrate-responsive regulator [39]. Moreover, the *OsPHR1, OsPHR2*, *OsPHR3* and *OsPHR4* participate in Pi-starvation signaling [33, 34, 40]. Our current study reveals that G2-like transcription factor also plays a critical role in regulating flowering time in rice.

### *OsPHL3* acts as a novel repressor of flowering under LDs and SDs

Currently, the cloned floral genes could be divided into two categories: day-length independent genes and day-length dependent genes. The first group is the constitutive flowering regulators regardless of day-length [8, 14–18, 25, 27, 28]. The second group encompasses genes that are functionally associated with day-length [9, 20, 22, 40–44].

Our current work identified *OsPHL3* as a constitutive repressor of flowering under both LDs and SDs. The *OsPHL3*-OE transgenic plants displayed a late-flowering phenotype with the genetic background of TP309, whereas the knockout mutants exhibit an early-flowering phenotype under two different day length conditions and two genetic backgrounds. Therefore, these results support that the function of *OsPHL3* was highly conserved between rice cultivars TP309 and Nipponbare.

In rice, there are two pathways to control the flowering time: one is the conserved *OsGI*-*Hd1*-*Hd3a* pathway and the other is the rice unique *Ghd7*-*Ehd1*-*Hd3a/RFT1* pathway. The diurnal expression pattern indicated that *OsPHL3* functions as a negative flowering regulator by inhibiting the expression of *RFT1* and *Hd3a*. This was achieved through promoting the expression of Hd1 under LDs, and through suppressing the expression of *Ehd1*/*Hd1* under SDs (Fig. 7). Thus, our data suggest that *OsPHL3* is involved in *Ehd1*- and *Hd1*-mediated regulatory pathway in rice. Whether *OsPHL3* directly regulated *Ehd1*, *Hd1* and other factors remains to be determined. Future work will aim to dissect the cis-regulatory elements directly bound by OsPHL3 on chromatin, and to identify functionally partner proteins interacting with *OsPHL3*.

## Conclusions

First, gene co-expression networks allowed us to identify *OsPHL3*, a new gene involved in regulating the flowering pathway in rice. Second, overexpression of *OsPHL3* delayed flowering time in rice under LD and SD conditions. Knockout of *OsPHL3* by genome-editing tool CRISPR/Cas9 resulted in earlier flowering regardless of day-length. Third, *OsPHL3* repressed flowering time by impacting *Ehd1*/*Hd1-*mediated pathway under SDs, and *Hd1*-mediated pathway under LDs. Our study indicates that gene co-expression network analysis is a powerful tool for identifying genes affecting flowering time in rice.

## Methods

### Plant materials and growth conditions

Japonica rice (*Oryza sativa* L.) variety Nipponbare and Taipei309 (TP309) were used in this study. Nipponbare is the day length-sensitive variety. All rice plants were grown in the field of Institute of Genetics and Developmental Biology, Chinese Academy of Sciences, in Hainan (18°30’N, 110°2′E) and Beijing (40°13’N, 116°13′E).

The rice plants for diurnal expression analysis were firstly sown in pots in the greenhouse. After growing under natural day-length conditions for 20 and 50 days, the rice plants grown in a controlled-growth chamber under controlled long day (CLD) (14 h light/10 h darkness) and controlled short day conditions (CSD) (10 h light/14 h darkness) conditions with light intensity of 800 umol.m^−2^s^−1^for additional 14 days. Leaf samples were collected from 34-day-old plants under CLDs and 64-day-old plants under CSDs. We collected leaves under CLDs and CSDs seedlings in 4 h interval for total RNA extraction.

### Bioinformatics analysis

The transcriptome data was downloaded from the website (https://www.ncbi.nlm.nih.gov/geo/query/acc.cgi?acc=GSE54274) (GSE54274_rice_leaf_section_fpkm.txt.gz) [33]. The 14-day-old rice leaves were cut into 11 parts, which represented the immature-to mature gradient of variation in gene expression [33, 45]. The family information of rice transcription factors were downloaded from Planttfdb (http://planttfdb.cbi.pku.edu.cn/) [46]. We calculated absolute value of the Pearson correlation coefficient between flowering time regulators and TFs using custom Perl script. The absolute value of the Pearson correlation coefficient greater than 0.8 between the flowering time regulators and TFs were listed in Additional file 1: Table S1. With the absolute value of Pearson correlation coefficient greater than 0.6, 28 G2-like family transcription factors co-expressed with flowering time genes (Fig. 1), which were constructed by Cytoscape v.2.8.3 [47]. Venn diagram was created using the online tool (http://bioinformatics.psb.ugent.be/webtools/Venn/).

All rice G2-like protein sequence were downloaded from Rice Genome Annotation Project (http://rice.plantbiology.msu.edu/); the *Arabidopsis* protein sequences of four G2-like sequences were downloaded from The Arabidopsis Information Resource (TAIR) (http://www.arabidopsis.org/). The alignment between rice and *Arabidopsis* was generated with ClustalW [48] using default setting (http://clustalw.ddbj.nig.ac.jp/). We used the software GeneDoc to perform the graphical output between OsPHL3 and AtPHL3 alignment. The neighbor-joining trees were constructed using MEGA software [49]. Domain prediction was carried out by Pfam database [50] (http://pfam.xfam.org/search/sequence).

### Vector construction and transformation

To construct the overexpression vector, the 750-bp coding region of *OsPHL3 (*LOC_Os09g12750) was amplified using KOD plus DNA polymerase (Toyobo, Japan). Gene specific primers, 5′-GAATTC ATGTTCCCGCCTGGGCTGATCCACCACCGGCC-3′.

5′-GGATCC CCGCAAGACTTGCGCTTAAGCTCAGGTGACTT- 3′ (*Eco*R I and *Bam*H I sites underlined) were used to amplify the coding sequence of *OsPHL3* using cDNA of Nipponbare as template. The digested PCR amplicons were cloned into the vector pEZR (K)-LN and confirmed by Sanger sequencing. The construct was introduced into the *Agrobacterium tumefaciens* LBA4404 by electroporation and subsequently transformed into the rice cultivar TP309 [51].

Positive transgenic plants were detected through the amplification of GFP in the vector and another fragment of *OsPHL3* using the forward primer *OsPHL3*-F 5′- CCCGAGAAAGCAACACCTAAAA-3′ and reverse primer GFP-R 5′- GTCGTCCTTGAAGAAGATGGTGC -3′. PCR amplification was carried out using the following profile: 95 °C for 5 min; followed by 32 cycles at 95 °C for 20 s; 58 °C for 30 s, 72 °C for 40 s; and final extension at 72 °C for 7 min.

We used CRISPR/Cas9 system to generate *osphl3* plants by the Biogle Company (Hangzhou, China) [52]. Two sgRNAs targeting *OsPHL3* CDS: sg37: 5′-GGGCGGCGAGGCGGGGCGT-3′, and sg93: 5′-GTCCCAAGCCCCGCCTCCGG-3′. Vector pBGK032 containing single sgRNA was introduced into rice cultivar TP309 and Nipponbare by *A. tumefaciens* strain EHA105. Each constructs obtained more than 14 independent transgenic lines. To confirm the successful mutants, the genome DNA was extracted from the T2 generation transgenic plants to amplify the target regions and Sanger sequencing. The primers used were listed in Additional file 5: Table S2.

### Subcellular localization of OsPHL3 protein

To detect the subcellular localization of OsPHL3 protein, full-length CDS of *OsPHL3* was fused to green fluorescent protein (GFP) driving by the 35S CaMV promoter. The mCherry-VirD2NLS vector (mCherry) was used as a nuclei marker. The protoplasts were isolated from 14-d-old rice seedlings and transformed with *OsPHL3-*pEZR (K)-LN. Fluorescence in the transformed protoplasts were visualized using confocal microscope (Zeiss Axio Imager.Z2) [53].

### Analysis of transcriptional activation

We used Yeastmaker Yeast Transformation System 2 (Clontech, USA) to analysis transcriptional activation potential. The full-length coding sequence, the N-terminal and C-terminal deletion were amplified with the primers (Additional file 5: Table S2) by PCR and cloned into pGBKT7 to fuse the GAL4 binding domain to create the plasmids BD-Myb domain-C term (1–251), BD-Myb domain (1–100) and BD-C term (101–251). The transformants were grown on -Trp SD media or –Trp/-His/−Ade SD media.

### RNA isolation and quantitative real-time PCR analysis

Total RNA was isolated from different tissues and organs using Trizol reagent (Invitrogen, USA). DNA digestion was treated with DNase (Takara, Japan), and first-strand cDNA was synthesized by SuperScript kit (Takara, Japan). The rice *ubiquitin* gene was used as an internal control and quantitative RT-PCR was performed using the Bio-Rad CFX96 real-time system. The procedure was: 95°Cfor 3 min and 42 cycles of 95 °C for 5 s, 60 °C for 10 s. The primers of photoperiodic flowering pathway genes (*OsGI*, *Hd1*, *Hd3a*, *RFT1*, *Ehd1*, *Ghd7*) were obtained as reported previously [54, 55]. Average values were obtained from three technical replicates and two biological replicates. The primers were listed in Additional file 5: Table S2.

## Additional files

Additional file 1: **Table S1.** The Pearson correlation coefficient value between flowering time regulators (*OsGI*, *Ghd7*, *OsMADS14* and *Hd3a)* and top-five transcription factor families*. (XLSX 19 kb)*

Additional file 2: **Figure S1.** Venn diagram illustrating shared and unique top-five transcription factor families between *OsGI*, *Ghd7*, *OsMADS14* and *Hd3a. (TIF 2261 kb)*

Additional file 3: **Figure S2.** (A) Expression analysis of *OsPHL3* in various tissues and organs in the variety of TP309 and Nipponbare. (B) Diurnal rhythm pattern of *OsPHL3* expression under CLDs in the variety of TP309. (C) Diurnal rhythm pattern of *OsPHL3* expression under CSDs in the variety of TP309. (TIF 58 kb)

Additional file 4: **Figure S3.** Diurnal expression patterns of *OsGI* and *Ghd7* in WT and *OsPHL3-*OE in the genetic background of TP309 under CLDs and CSDs by qRT-PCR analysis. (TIF 107 kb)

Additional file 5: **Table S2.** The sequence of primers used in this study. (DOCX 13 kb)

## Abbreviations

CLD: controlled long-day
CLDs: controlled long-day conditions
*CO*: *CONSTANS*
CSD: controlled short-day
CSDs: controlled short-day conditions
*DTH8*: *Days to heading 8*
*Ehd1*: *Early heading date 1*
FT: *FLOWERING LOCUS T*
*Ghd7*: *Grain number, plant height and heading date 7*
*GI*: *GIGANTEA*
*Hd1*: *Heading date 1*
*Hd3a*: *Heading date 3a*
LD: Long-day
LDs: Long-day conditions
*OsPHL3-OE*: *OsPHL3-*overexpression
*RFT1*: *RICE FLOWERING LOCUS T 1*
SD: Short-day
SDs: Short-day conditions
WT: Wild-type
