# Supplementary figures and images for: Identification of a G2-like transcription factor, OsPHL3, functions as a negative regulator of flowering in rice by co-expression and reverse genetic analysis
Source: BMC Plant Biol. 2018 Aug 6;18:157. doi: 10.1186/s12870-018-1382-6 (PMC6091178; doi:10.1186/s12870-018-1382-6)

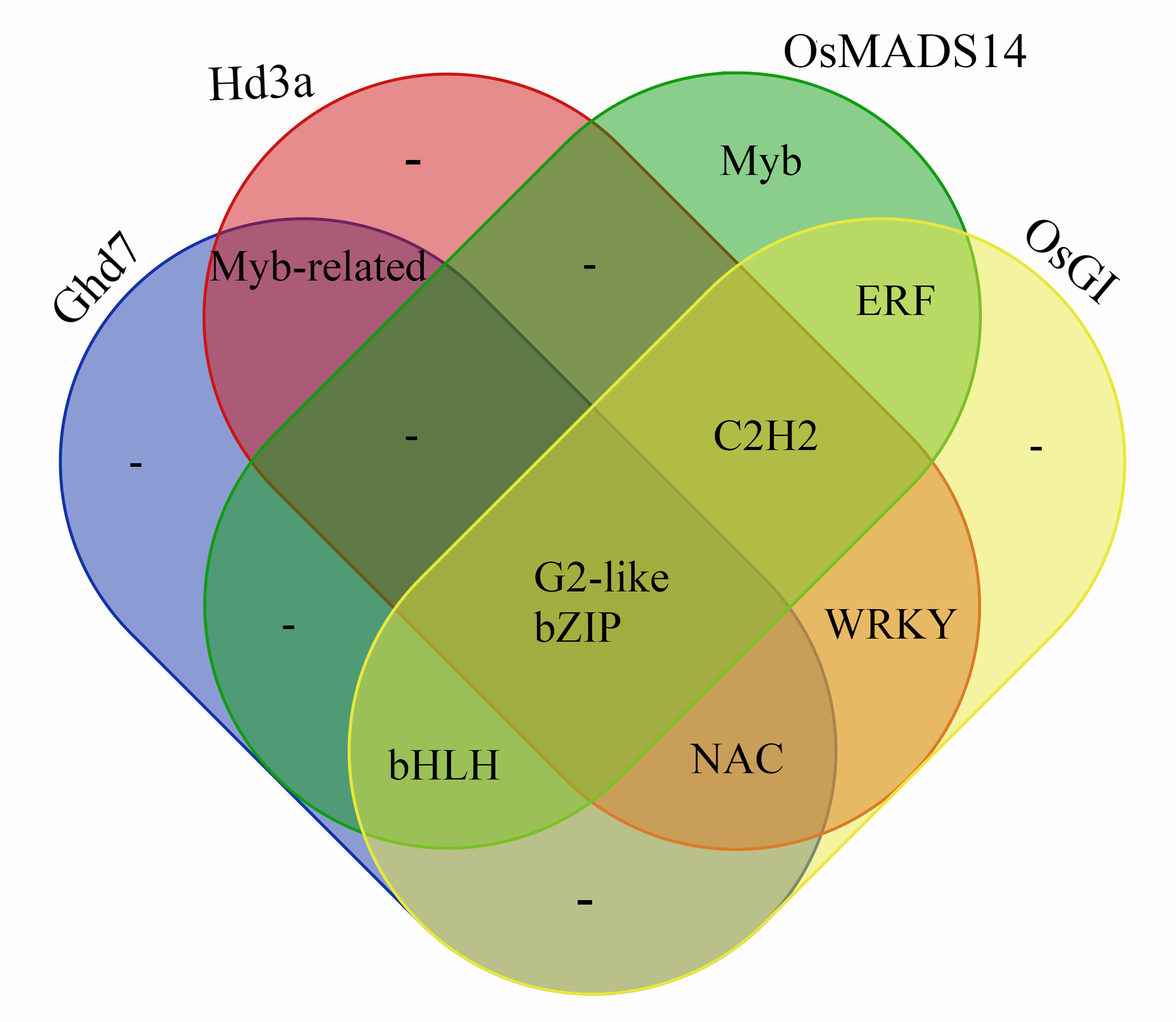

Supplement: Supplementary file 2 — Figure S1. Venn diagram illustrating shared and unique top-five transcription factor families between OsGI, Ghd7, OsMADS14 and Hd3a. (TIF 2261 kb) [file 12870_2018_1382_MOESM2_ESM.tif]

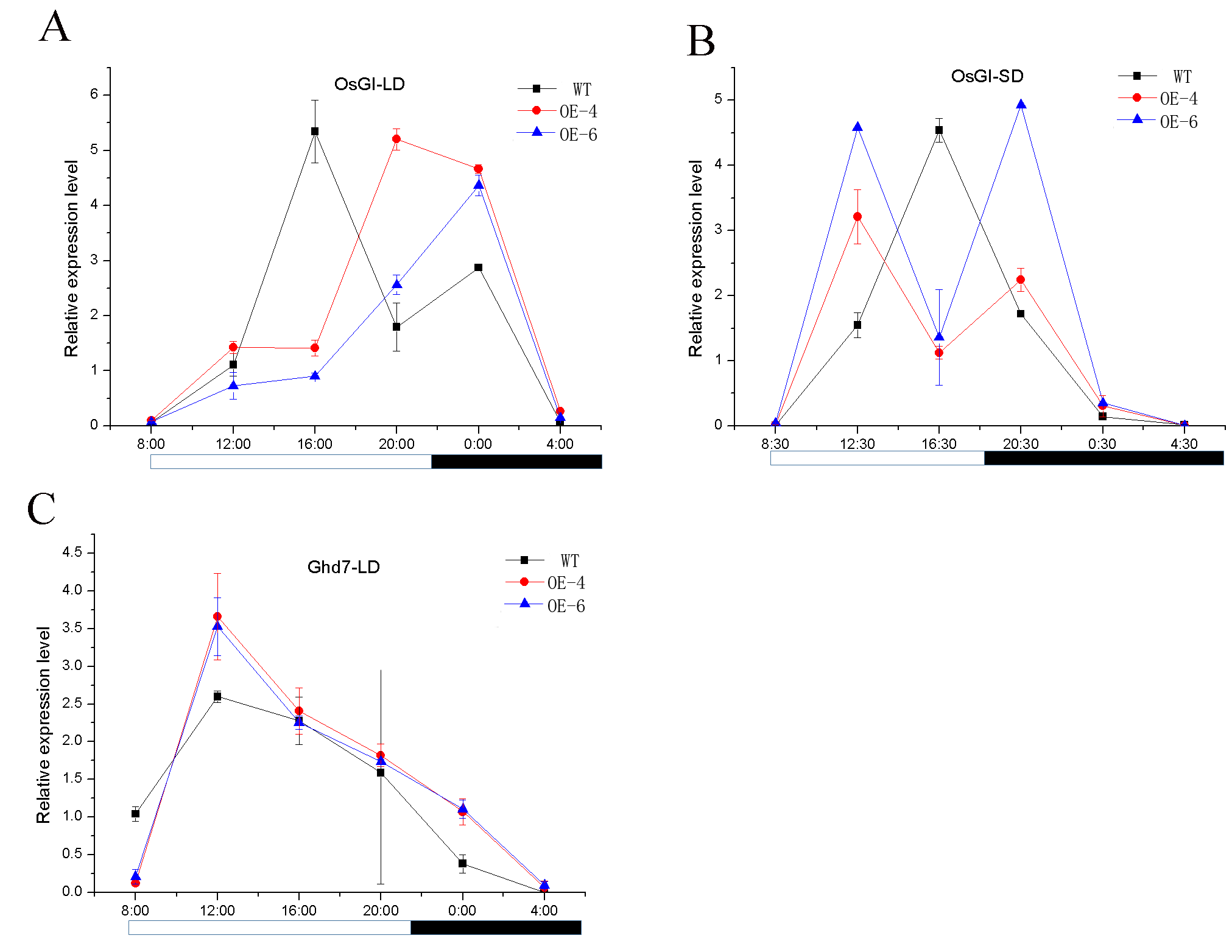

Supplement: Supplementary file 4 — Figure S3. Diurnal expression patterns of OsGI and Ghd7 in WT and OsPHL3-OE in the genetic background of TP309 under CLDs and CSDs by qRT-PCR analysis. (TIF 107 kb) [file 12870_2018_1382_MOESM4_ESM.tif]
