# Supplementary material for: Identification of a G2-like transcription factor, OsPHL3, functions as a negative regulator of flowering in rice by co-expression and reverse genetic analysis
Source: BMC Plant Biol. 2018 Aug 6;18:157. doi: 10.1186/s12870-018-1382-6 (PMC6091178; doi:10.1186/s12870-018-1382-6)
Supplement: Supplementary file 5 — Table S2. The sequence of primers used in this study. (DOCX 13 kb) [file 12870_2018_1382_MOESM5_ESM.docx]

**Additional file 5:Table S2. The sequence of primers used in this study**

| **Primer name** | **Sequence (5′- 3′)** | **Description** |
| --- | --- | --- |
| sg356-seq(ge)-F | GAGCAGCAAAGGTCTCGTCT | Primers for identification  *osphl3-1* mutants |
| sg356-seq(ge)-R | AAATCTCCGCTCCCAATCCG |  |
| sg 412-seq(ge)-F | GAGCAGCAAAGGTCTCGTCT | Primers for identification  *osphl3-2* mutants |
| sg 412-seq(ge)-R | AAATCTCCGCTCCCAATCCG |  |
| LN7YF1 | CATG GAGGCCGAATTCA  TGTTCCCGCCTGGGCTGA | The forward primer for constructing either PLN7 vector or PLN7N1-100 vector |
| LN7YR1 | GCAGGTCGACGGATCCTC  AGCAAGACTTGCGCTTAA | The reverse primer for constructing either PLN7 vector or PLN7C101-251vector |
| LN7YR2 | GCAGGTCGACGGATCCT  CATTTAGATTGCTCCGATGC | The reverse primer for constructing PLN7N1-100 vector |
| LN7YF2 | CATG GAGGCCGAATTC  GATGCATCCTATCTTCTAG | The forward primer for constructing PLN7C101-251 vector |
| *OsGI*-F | GTGGATGCGCTTTGTGACAT | *OsGI* primers |
| *OsGI*-R | GGCCTGCAGAACGATAGCA |  |
| *Ghd7*-F | AGGTGCTACGAGAAGCAAATCC | *Ghd7* primers |
| *Ghd7*-R | GGGCCTCATCTCGGCATAG |  |
| *Hd1*-F | TCAGCAACAGCATATCTTTCTCATCA | *Hd1* primers |
| *Hd1*-R | TCTGGAATTTGGCATATCTATCACC |  |
| *Ehd1*-F | GGATGCAAGGAAATCATGGA | *Ehd1* primers |
| *Ehd1*-R | AATCCCATCGGAAATCTTGG |  |
| *Hd3a*-F | CTTCAACACCAAGGACTTCGC | *Hd3a* primers |
| *Hd3a*-R | TAGTGAGCATGCAGCAGATCG |  |
| *RFT1*-F | TGACCTAGATTCAAAGTCTAATCCTT | *RFT1* primers |
| *RFT1*-R | TGCCGGCCATGTCAAATTAATAAC |  |
| *OsPHL3*-F | CCGAGAAAGCAACACCTAAAAC | *OsPHL3* primers |
| *OsPHL3*-R | CTTTTCCAGATTGTTTCCCCAG |  |
| *Ubq*-F | AACCAGCTGAGGCCCAAGA | *Ubiquitin* primers |
| *Ubq*-R | ACGATTGATTTAACCAGTCCATGA |  |
